# Transfer of Tactile Sensors Using Stiction Effect Temporary Handling

**DOI:** 10.3390/mi12111330

**Published:** 2021-10-29

**Authors:** Peng Zhong, Ke Sun, Chaoyue Zheng, Heng Yang, Xinxin Li

**Affiliations:** 1 State Key Laboratory of Transducer Technology, Shanghai Institute of Microsystem and Information Technology, Chinese Academy of Sciences, Shanghai 200050, China; pengzhong@mail.sim.ac.cn (P.Z.); sunke@mail.sim.ac.cn (K.S.); osananajimi@mail.sim.ac.cn (C.Z.); xxli@mail.sim.ac.cn (X.L.); 2 School of Microelectronics, University of Chinese Academy of Sciences, Beijing 100049, China

**Keywords:** stiction effect, temporary handling, SETH, CMOS, MEMS, tactile sensor, stiction-contact, Au–Si eutectic, flip-chip

## Abstract

A novel method for transfer of tactile sensors using stiction effect temporary handling (SETH) is presented to simplify the microelectromechanical-system (MEMS)/CMOS integration process, improve the process reliability and electrical performance, and reduce material constriction. The structure of the tactile sensor and the reroute substrate were first manufactured separately. Following the release process, the stiction-contact structures, which are designed to protect the low-stress silicon nitride diaphragm of the tactile sensor and prevent the low-stress silicon nitride diaphragm from moving during the subsequent bonding process, are temporarily bonded to the substrate owing to the stiction effect. After the released tactile sensor is bonded to the reroute substrate by Au–Si eutectic flip-chip bonding, a pulling force perpendicular to the bonded die is applied to break away the temporary supported beam of the tactile sensor, and the tactile sensor is then successfully transferred to the reroute substrate. The size of the transferred tactile sensor is as small as 180 μm × 180 μm × 1.2 μm, and the force area of the tactile sensor is only 120 μm × 120 μm × 1.2 μm. The maximum misalignment of the flip-chip bonding process is approximately 1.5 μm. The tactile sensors are tested from 0 to 17.1 kPa when the power supply is 5 V, resulting in a sensitivity of 0.22 mV/V/kPa, 0.26 mV/V/kPa, 0.27 mV/V/kPa and 0.27 mV/V/kPa, separately. The stress caused by the Au–Si eutectic flip-chip bonding ranges from −5.83 to +5.54 kPa. The temporary bonding strength caused by stiction is calculated to be larger than 7.06 kPa and less than 22.31 kPa. The shear strength of the bonded test structure is approximately 30.74 MPa and the yield of the transferred tactile sensors is as high as 90%.

## 1. Introduction

Numerous efforts have been made to integrate microelectromechanical systems (MEMS) and complementary metal-oxide-semiconductor (CMOS) devices [1,2,3,4,5,6,7,8,9]. There are two approaches for integrating MEMS sensors and actuators with CMOS devices. The first is monolithic integration, where the MEMS and CMOS devices are fabricated on the same silicon substrate using a dedicated fabrication process [10]. The second approach is hybrid integration, in which the MEMS and CMOS devices are assembled using chip-interconnection methods (i.e., tape automated bond (TAB), wire bond, and flip-chip bond) [11,12,13,14,15,16].

Monolithic integration has been widely studied owing to its advantages of lower electronic parasitics, reduced chip pinout, and smaller size. There are three methods for monolithic integration: pre-CMOS, intra-CMOS, and post-CMOS [17,18,19,20]. The classification of the three approaches is based on the sequence of fabrication of the MEMS and CMOS devices. In the pre-CMOS and intra-CMOS approaches, contamination issues should be considered after the wafer has undergone some proprietary MEMS processes [6]. Thus, dedicated fabrication is required for the pre-CMOS and intra-CMOS. Moreover, the thermal budget should be considered in the intra-CMOS approach because of the high-temperature front-end-of-line processes [10]. In the post-CMOS approach, materials and processes used for the fabrication of MEMS devices must be carefully considered to avoid damage to the completed CMOS device during MEMS fabrication processes [21].

Hybrid integration, which enables the MEMS and CMOS devices to be fabricated independently, is currently the most common approach for MEMS and CMOS integration because of its shorter development time, lower cost, flexible material selection, and simpler fabrication process [22]. In hybrid integration technologies, flip-chip bond provides the highest packaging density, better reliability, and better electrical and thermal performance. However, with the development of advanced packaging technologies (i.e., three-dimensional packaging technology) and advanced microsystems (i.e., radio frequency devices), the average wire length of the flip-chip bond becomes significant, which can cause higher electrical parasitics between the MEMS and CMOS devices [23].

A modified flip-chip method for the transfer of released microstructures has been proposed by Singh et al. [13]. In this modified method, two types of MEMS structure are fabricated and released, followed by the fabrication of a target die, which is patterned with metal bumps. Then, the released MEMS structures and the target die are bonded by cold welding the indium solder to the copper. Finally, the bonded structure is carefully separated to break the tethers of the MEMS structures, thereby transferring the MEMS structures onto the target die. This modified method enables the MEMS and CMOS devices to be fabricated separately, simplifying the fabrication process and providing flexibility in the selection of the MEMS/CMOS process and substrate. Moreover, the signal path can be shortened, which improves the electronic performance of the integrated device. However, the released MEMS microstructures, only supported by polysilicon tethers, are at risk of being damaged by shear forces during the bonding and transferring processes. Moreover, the displacement of MEMS structures caused by shear force decreases the alignment precision of the flip-chip bonding.

In this study, a novel method for transfer of tactile sensors using stiction effect [24] temporary handling (SETH) is presented to simplify the MEMS/CMOS integration process, improve the process reliability and electrical performance, and reduce material constriction. The tactile sensor and reroute substrate are first manufactured separately. Then, the tactile sensor is released and placed into deionized (DI) water for 24 h to bond the stiction-contact structures temporarily to the substrate through the stiction effect, thereby avoiding the damage and movement of the diaphragm of the tactile sensor during subsequent flip-chip bonding. Next, the released tactile sensor is bonded to the reroute substrate by Au–Si eutectic flip-chip bonding. Finally, a pulling force perpendicular to the bonded die is applied to break away the temporary supported beam of the tactile sensor, and the tactile sensor is then transferred to the reroute substrate. The size of the transferred tactile sensor is as small as 180 μm × 180 μm × 1.2 μm, and the force area of the tactile sensor is only 120 μm × 120 μm × 1.2 μm. The maximum misalignment of the flip-chip bonding process is approximately 1.5 μm. The stress caused by the Au–Si eutectic flip-chip bonding is from −5.83 to +5.54 kPa. The tactile sensors are tested from 0 to 17.1 kPa when the power supply is 5 V, resulting in a sensitivity of 0.22 mV/V/kPa, 0.26 mV/V/kPa, 0.27 mV/V/kPa and 0.27 mV/V/kPa, separately. The shear strength of the bonded test structure is approximately 30.74 MPa and the yield of the transferred tactile sensors is as high as 90%.

## 2. Design Principle

**Figure 1:**
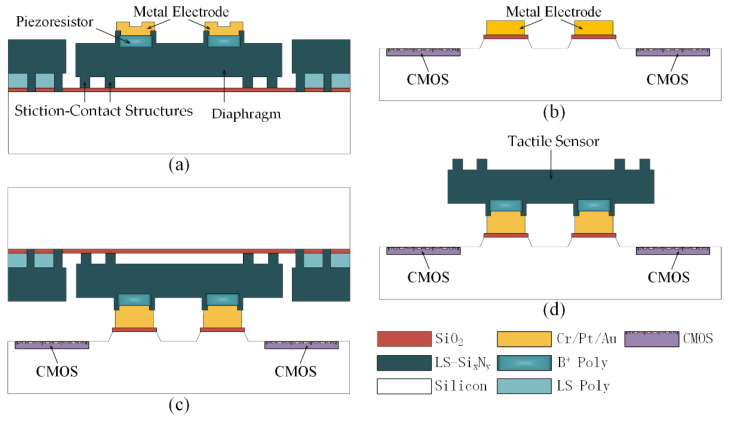
Design process flow of complementary metal-oxide-semiconductor (CMOS)-compatible batch transfer of tactile sensor using stiction effect temporary handling (SETH). (**a**) Tactile sensor is manufactured, and the stiction-contact structures are temporary bonded to the substrate; (**b**) CMOS device and the metal electrode used for bonding are fabricated; (**c**) MEMS tactile sensor is bonded to the CMOS device; (**d**) microelectromechanical system (MEMS) tactile sensor is transferred to the CMOS device by applying a pulling force perpendicular to the bonded device.

The principle of CMOS-compatible batch transfer of tactile sensors using SETH is shown in Figure 1. First, the tactile sensor is manufactured, and the stiction-contact structures, which are designed to protect the low-stress silicon nitride (LS-Si_x_N_y_) diaphragm of the tactile sensor, are temporarily bonded to the thermal oxide layer owing to the stiction effect, as shown in Figure 1a. Next, the CMOS device and metal electrode used for bonding are fabricated, as shown in Figure 1b. Thereafter, the MEMS tactile sensor is bonded to the CMOS device, as shown in Figure 1c. Finally, the MEMS tactile sensor is transferred to the CMOS device by applying a pulling force perpendicular to the bonded device, as shown in Figure 1d.

### 2.1. Design of Tactile Sensor

**Figure 2:**
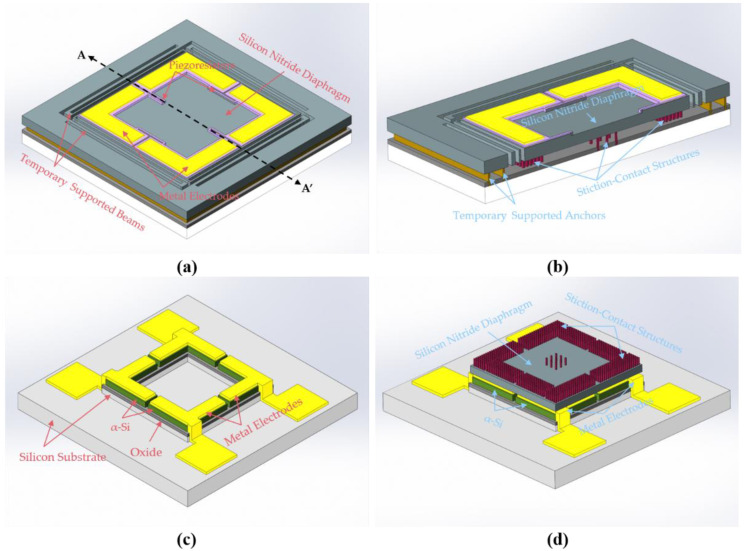
(**a**) Schematic of the tactile sensor; (**b**) Cross-section cut along the line A–A′ in (**a**); (**c**) Schematic of the reroute substrate; (**d**) Schematic of the transferred device.

**Figure 3:**
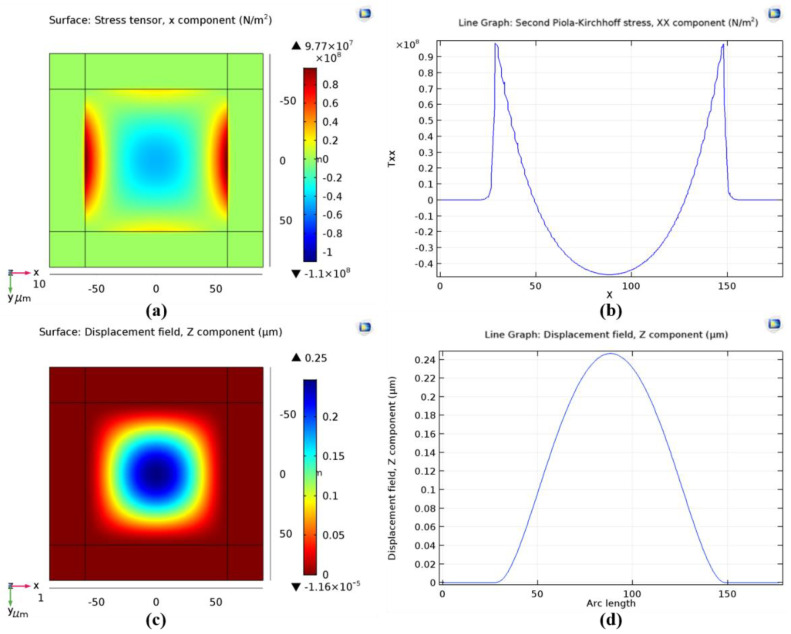
COMSOL simulation results of tactile sensor. (**a**) Stress simulation of the silicon nitride diaphragm; (**b**) Stress simulation result shows that the maximum stress occurs at the edge of the silicon nitride diaphragm, and its value is 98.59 MPa; (**c**) Displacement simulation of the silicon nitride diaphragm; (**d**) Displacement simulation result shows that the maximum displacement occurs at the center of the silicon nitride diaphragm, and its value is 0.25 μm.

To verify the principle of CMOS-compatible batch transfer of tactile sensors using SETH, a tactile sensor with a low-stress silicon nitride diaphragm was designed for transfer to the reroute substrate. The process flow is similar to that shown in Figure 1. Figure 2a shows a schematic of the designed tactile sensor, and Figure 2b shows a cross-sectional cut of the tactile sensor along line A–A′. Figure 2c shows a schematic of the reroute substrate, and Figure 2d shows a schematic of the transferred tactile sensor. As shown in Figure 2a, the dimensions of the low-stress silicon nitride diaphragm are 120 μm × 120 μm × 1.2 μm, and four p-type resistors are formed on the edge of the silicon nitride diaphragm surface, where the component of the stress tensor in silicon nitride diaphragm is found to be largest through simulation using COMSOL, as shown in Figure 3. The size of the designed transferred tactile sensor is 180 μm × 180 μm × 1.2 μm and the center diaphragm of the tactile sensor is 120 μm × 120 μm × 1.2 μm, the area outside the center diaphragm is designed for Au-Si eutectic bonding, as shown in Figure 3a,c. As the diaphragm of the tactile sensor is fabricated by LPCVD isotropic low-stress silicon nitride, the density, Young’s modulus, and Poisson’s ratio of the low-stress silicon nitride used for COMOSL simulation are set as 3000 kg/m^3^, 360.5 GPa and 0.24 [25], separately. Figure 3b shows that the largest stress is approximately 98.59 MPa under 35 kPa pressure, which occurs at the edge of the silicon nitride diaphragm. Figure 3d shows that the maximum displacement occurs at the center of the silicon nitride diaphragm, and its value is approximately 0.25 μm. The four piezo resistors are connected using heavily doped polysilicon to form a flat surface, and a composite metal layer with a flat surface is then deposited on the heavily doped polysilicon to a form the Wheatstone bridge and the metal electrodes designed for Au-Si eutectic bonding, as shown in Figure 2a,b. The composite metal layer with a flat surface increases the bonding area of metal electrodes, thereby improving the bonding strength of Au-Si eutectic bonding process. The sensitivity of the tactile sensor is

(1) $$S=\frac{\Delta V}{\Delta P}=\frac{\pi_{44}a^{2}}{4h^{2}}V_{\mathrm{cc}}$$

where Δ*V* and Δ*P* are the changes in the output voltage of the Wheatstone bridge and the pressure of the silicon nitride diaphragm, respectively, $\pi_{44}$ is the piezoresistive coefficient of polysilicon, *a* is the half side length of the silicon nitride diaphragm, and *h* is the thickness of the silicon nitride diaphragm. Theoretically, the sensitivity of the tactile sensor is 2.55 mV/kPa when the power supply is 5 V.

### 2.2. Design of Stiction-Contact Structures

Previous studies have suggested that electrostatic forces, van der Waals forces, or surface tension may be responsible for sticking or stiction [26,27], which causes the permanent attachment of slender surface micromachined structures to the underlying substrate after drying [28]. To conceptualize the scale of the stiction forces, the three causes are each illustrated using an ideal system consisting of two smooth parallel surfaces with separation d and shared area S.

The electrostatic force results from electrostatic charging or differences in the work functions of the two smooth parallel surfaces, and the work function differences yield at most 1 V potentials in equilibrium [26]. Neglecting the internal space charge regions, the electrostatic force, $F_{\mathrm{EL}}$, is [26]

(2) $$F_{\mathrm{EL}}=\frac{\varepsilon_{0}U^{2}}{2d^{2}}\cdot S$$

where $\varepsilon_{0}$ and U are the relative permittivity of the air gap and the potential difference between the two parallel surfaces, respectively.

The van der Waals force results from the interaction between the instantaneous dipole moment of atoms. The expression for the van der Waals force, $F_{\mathrm{VDW}}$, can be expressed as [29]:

(3) $$F_{\mathrm{VDW}}=\frac{A}{6\pi d^{3}}\cdot S$$

where A is the Hamaker constant. For the SiO_2_–air–SiO_2_ case, the Hamaker constant is $5.4\times 10^{-20}$ J [30].

The surface tension of the diminishing liquid induces an attractive capillary force during the drying of capillary liquids trapped in the two parallel surfaces, and the capillary force, $F_{\mathrm{CF}}$, can be described as [26]

(4) $$F_{\mathrm{CF}}=\frac{\gamma \left(\cos\theta_{1}+\cos\theta_{2}\right)}{d}\cdot S$$

where *γ* is the surface tension (73 mN/m for water), and $\theta_{1}$ and $\theta_{2}$ are the contact angles between the two parallel surfaces.

As Equations (2)–(4) show, when the distance between two smooth parallel surfaces is larger than 10 nm, the electrostatic force and van der Waals force can be negligible compared with the surface tension; thus, the total adhesion forces between two smooth parallel surfaces can be simplified as

(5) $$F_{\mathrm{Total}\_\mathrm{AF}}=F_{\mathrm{EL}}+F_{\mathrm{VDW}}+F_{\mathrm{CF}}\approx \frac{\gamma \left(\cos\theta_{1}+\cos\theta_{2}\right)}{d}\Delta S$$

To ensure that the tactile sensor can be successfully transferred to the reroute substrate, as shown in Figure 2d, the temporary bonding strength caused by stiction should be less than the bonding strength of the flip-chip bonding. As Equation (5) shows, the total adhesion forces can be decreased by reducing the shared area between two parallel surfaces. Therefore, different areas of stiction-contact structures (from 2916 to 9000 ${\mu m}^{2}$) are designed to reduce the shared area between the silicon nitride diaphragms of the tactile sensor and substrate. A cross-sectional schematic view of the stiction-contact structures is shown in Figure 2b. In addition, the stiction-contact structures can prevent the silicon nitride diaphragm from moving in the subsequent bonding process because of its temporary bonding to the substrate, thereby improving the alignment precision of flip-chip bonding.

To ensure that the deadhesion process does not damage the bonding strength of the transferred device, the Au–Si eutectic bonding technique was employed to ensure the bonding strength of the subsequent flip-chip bonding process, in which the bonding strength is usually larger than 16 MPa in the laboratory [31]. Moreover, the metal electrode area for Au–Si eutectic bonding is designed to be 12,320 ${\mu m}^{2}$, which is much larger than the designed area of stiction-contact structures, so that the adhesion forces caused by stiction can be negligible compared with the Au–Si eutectic bonding strength.

**Figure 4:**
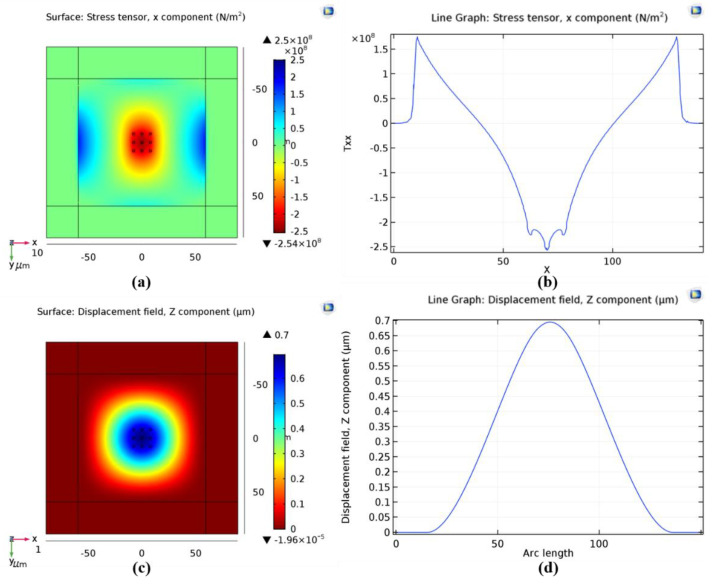
COMSOL simulation results of the stress and displacement of silicon nitride diaphragm caused by stiction effect. (**a**,**b**) Maximum stress occurs at the edge of the silicon nitride diaphragm, and its value is approximately 167.71 MPa; (**c**,**d**) Maximum displacement occurs at the center of the silicon nitride diaphragm, and its value is approximately 0.69 μm.

As Equations (2)–(5) show, the total adhesion forces can be decreased by increasing the distance between two parallel surfaces. When the designed distance between the stiction-contact structures and substrate is 200 nm, the corresponding temporary bonding strength can be calculated as 0.73 MPa in theory. Assuming that the stiction occurs at the center of the silicon nitride diaphragm and the temporary bonding strength caused by stiction is 10 MPa, which is much larger than 0.73 MPa, the stress and displacement of the silicon nitride diaphragm are simulated by COMSOL (5.3, COMSOL, Inc., Burlington, Mam, USA), as shown in Figure 4. As the stiction-contact structures of tactile sensor are fabricated by LPCVD isotropic low-stress silicon nitride, the density, Young’s modulus, and Poisson’s ratio of the stiction-contact structures in COMSOL simulation are set as 3000 kg/m^3^, 360.5 GPa and 0.24 [25], separately. The designed dimensions of the stiction-contact structures in the center of low-stress silicon nitride diaphragm are 2 μm × 2 μm × 1.2 μm, as shown in Figure 4a,c. As Figure 4b shows, the maximum stress distributed at the edge of the silicon nitride diaphragm, and its value is approximately 167.71 MPa under 10 MPa pressure. Therefore, the silicon nitride diaphragm is not damaged by stiction because the fracture strength of the low-pressure chemical vapor deposition (LPCVD) silicon nitride (6.9 GPa at 298 K) [25] is much higher than 167.71 MPa. Figure 4d shows that the maximum displacement occurs at the center of the silicon nitride diaphragm, and its value is approximately 0.25 μm.

### 2.3. Design of Test Structures

**Figure 5:**
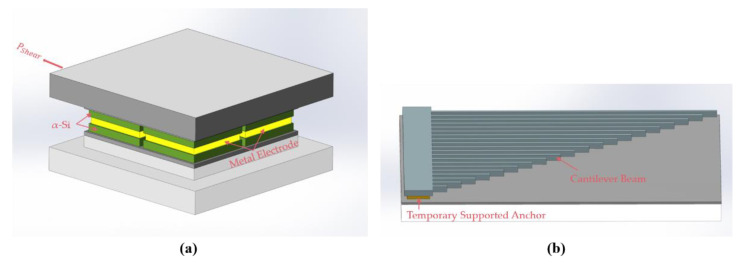
(**a**) Schematic of Au–Si eutectic bonding strength estimated by shear force test; (**b**) Prototype of 20 different types of cantilever beam designed to estimate the magnitude of the temporary bonding strength caused by stiction.

Metal electrodes with a width of 22 μm and a length of 81 μm were designed to estimate the Au–Si eutectic bonding strength of the transferred device, and the schematic of the test structure is similar to that shown in Figure 2c. After the test structures have been bonded by the Au–Si eutectic bonding technique, the bonding strength can be estimated by a shear force test, as shown in Figure 5a. In addition, 20 different types of cantilever beam with a width of 3 μm and a 20–400 μm length were designed to estimate the magnitude of the temporary bonding strength caused by stiction, as shown in Figure 5b. When the tips of the cantilever beams are fixed on the substrate after drying, the minimum temporary bonding strength caused by the stiction can be estimated as

(6) $$F_{\mathrm{TBS}\_S}=\frac{2Et^{3}}{3L^{3}}d$$

where E is the Young’s modulus of the cantilever beam material, 260.5 GPa at 298 K for LPCVD silicon nitride [24]; t and L are the thickness and length of the cantilever beam, respectively; and d is the distance between the cantilever beam and the substrate.

## 3. Fabrication

**Figure 6:**
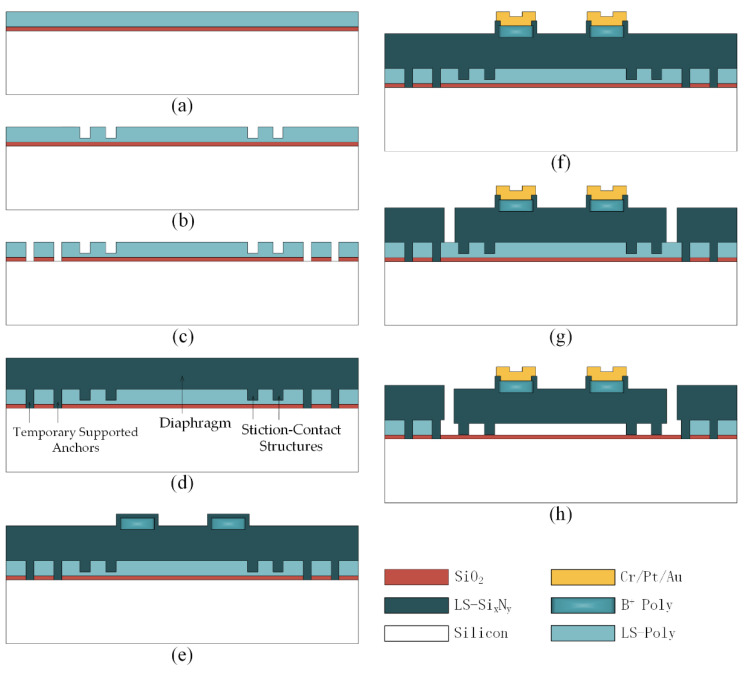
Process flow of the tactile sensor with a silicon nitride diaphragm. (**a**) A 450 nm-thick SiO_2_ layer is thermally grown to protect the substrate, followed by the deposition of 800 nm-thick sacrificial layer; (**b**) etching and LPCVD deposition to determine the distance between the stiction-contact structures and the substrate; (**c**) etching to form the channel for temporary supported anchors; (**d**) formation of the diaphragm of the tactile sensor, stiction-contact structures, and temporary supported anchors; (**e**) the 200 nm-thick low-stress silicon nitride layer is deposited after the formation of the piezoresistors; (**f**) a composite metal layer of Cr/Pt/Au is sputtered and patterned on the piezoresisitors after the contact windows of the piezoresisitors are formed; (**g**) formation of the release channel of the polysilicon sacrificial layer; (**h**) the XeF_2_ etching technique is employed to remove the polysilicon sacrificial layer, followed by the stiction-contact structures temporarily bonded to the substrate using the stiction effect.

The process flow of the tactile sensor with a silicon nitride diaphragm is depicted in Figure 6, and the detailed fabrication process is described below.

(a) A SiO_2_ layer with a thickness of 450 nm is thermally grown to protect the substrate from being damaged by the subsequent etching and release processes. Then, a layer of 800 nm-thick low-stress polysilicon is deposited as the sacrificial layer of the tactile sensor by the LPCVD technique, as shown in Figure 6a.

(b) The polysilicon is etched to the SiO_2_ layer using the deep reactive ion etching (DRIE) technique. Then, a layer of 200 nm-thick low-stress polysilicon is deposited by the LPCVD technique, and its thickness determines the distance between the stiction-contact structures and the substrate, as shown in Figure 6b.

(c) The 1 μm-thick low-stress polysilicon is etched using the DRIE technique. The 450 nm-thick SiO_2_ layer is subsequently etched to the silicon substrate by the reactive ion etching (RIE) technique, as shown in Figure 6c.

(d) A low-stress silicon nitride layer with a thickness of 1 μm is deposited by the LPCVD technique to form the diaphragm of the tactile sensor, stiction-contact structures, and temporary supported anchors of the silicon nitride diaphragm, as shown in Figure 6d.

(e) A 300 nm-thick LPCVD low-stress polysilicon layer is deposited and heavily doped by boron implantation, followed by the DRIE technique to form the piezoresistors of the tactile sensor. Next, a low-stress silicon nitride layer with a thickness of 200 nm is deposited by the LPCVD technique to protect the piezoresistors, as shown in Figure 6e.

(f) The 200 nm-thick low-stress silicon nitride layer is etched using the RIE technique to form the contact windows of the piezoresistors. Then, a composite metal layer of Cr/Pt/Au is sputtered and patterned on the piezoresistors, as shown in Figure 6f. The Pt layer of Cr/Pt/Au prevents the Au–Si alloy formed by the subsequent Au–Si eutectic flip-chip bonding process from penetrating the metal pads. The thicknesses of the Cr, Pt, and Au are 50 nm, 100 nm, and 300 nm, respectively.

(g) The release channel of the polysilicon sacrificial layer is formed by the DRIE technique after the 1.2 μm-thick low-stress silicon nitride layer is etched to the polysilicon sacrificial layer, as shown in Figure 6g.

(h) The XeF_2_ etching technique is employed to remove the polysilicon sacrificial layer. The released device is then placed in DI water for 24 h and dried at room temperature for 24 h to bond the stiction-contact structures temporarily to the substrate using the stiction effect, as shown in Figure 6h.

**Figure 7:**
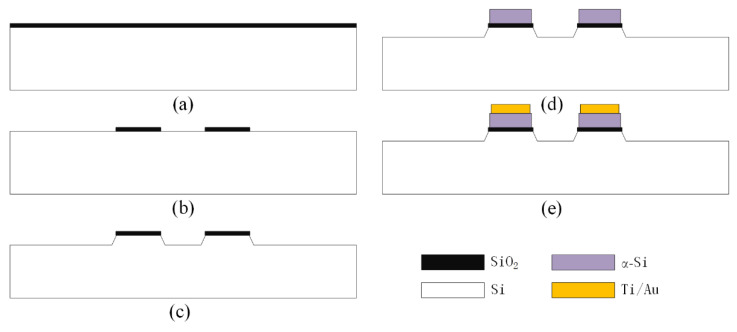
Process flow of the reroute substrate. (**a**) Oxidation to form the hard mask; (**b**) formation of the etching window of the KOH; (**c**) formation of the convex structure with a height of 7 μm; (**d**) deposition of the 1 μm-thick layer of amorphous silicon (α-Si) used for subsequent Au–Si eutectic bonding; (**e**) formation of the composite metal Ti/Au layer used to reroute the tactile sensor.

The process flow of the reroute substrate is shown in Figure 7, and the detailed fabrication process is described below.

(a) A SiO_2_ layer with a thickness of 200 nm is thermally grown as the hard mask in the following KOH wet etching process, as shown in Figure 7a.

(b) The 200 nm-thick SiO_2_ layer is etched using the RIE technique to expose the etching window of the KOH, as shown in Figure 7b.

(c) A convex structure with a height of 7 μm is formed during the KOH wet etching process to provide a space for the tactile sensor after bonding, as shown in Figure 7c.

(d) A 1 μm-thick layer of amorphous silicon (α-Si) is deposited by plasma-enhanced chemical vapor deposition (PECVD) for subsequent Au–Si eutectic bonding, as shown in Figure 7d.

(e) Finally, a composite metal Ti/Au layer is sputtered on the α-Si layer to reroute the tactile sensor, in which the Ti layer is used to decompose the native oxide on the surface of the α-Si. The thicknesses of the Ti and Au are 50 nm and 400 nm, respectively, as shown in Figure 7e.

**Figure 8:**
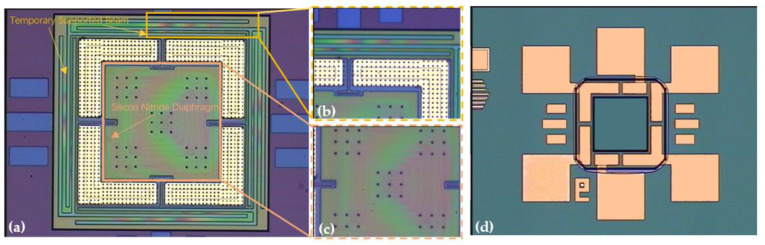
Optical microscope view of fabricated tactile sensor and rerouted substrate. (**a**) Optical microscope view of the fabricated tactile sensor; (**b**) enlarged optical microscope view of the temporary supported beam of the fabricated tactile sensor; (**c**) enlarged optical microscope view of the silicon nitride diaphragm of the tactile sensor; (**d**) optical microscope view of the fabricated reroute substrate.

An optical microscope view of the fabricated tactile sensor and reroute substrate is shown in Figure 8. The optical microscope view of the fabricated tactile sensor is shown in Figure 8a, and enlarged views of the temporary supported beam and the silicon nitride diaphragm of the tactile sensor are shown in Figure 8b,c, respectively. An optical microscope view of the fabricated reroute substrate is shown in Figure 8d. As Figure 8a–c show, the temporary supported beam and the silicon nitride diaphragm are colored under a microscope owing to the thin-film interference phenomenon, and the thin-film interference phenomenon is caused by the stiction, which temporarily bonds the temporary supported beam and the stiction-contact structures under the silicon nitride diaphragm to the substrate.

**Figure 9:**
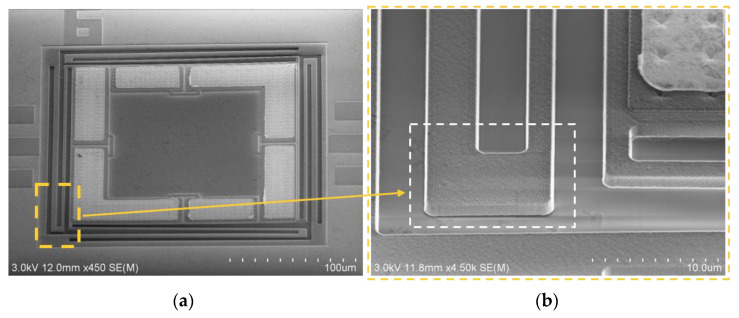
Scanning electron microscopy (SEM) view of the temporary supported beam of the fabricated tactile sensor. (**a**) SEM view of the fabricated tactile sensor; (**b**) enlarged view of the temporary supported beam shown in (**a**).

To analyze the stiction of the fabricated tactile sensor further, scanning electron microscopy (SEM) was employed. The SEM view of the temporary supported beam of the fabricated tactile sensor is shown in Figure 9. Figure 9b shows an enlarged view of the temporary supported beam shown in Figure 9a. As the Figure shows, the temporary supported beam is temporarily bonded to the substrate owing to the stiction effect.

## 4. Results and Discussion

**Figure 10:**
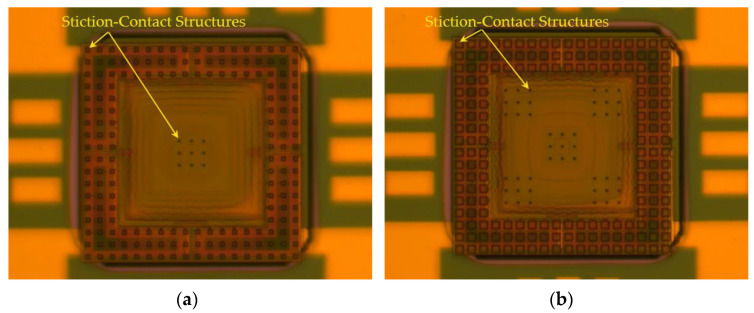
Optical microscopic view of the transferred devices. (**a**) Transferred device with minimum areas (2916$\;{\mu m}^{2}$) of stiction-contact structures; (**b**) Transferred device with maximum areas (9000 ${\mu m}^{2}$) of stiction-contact structures.

**Figure 11:**
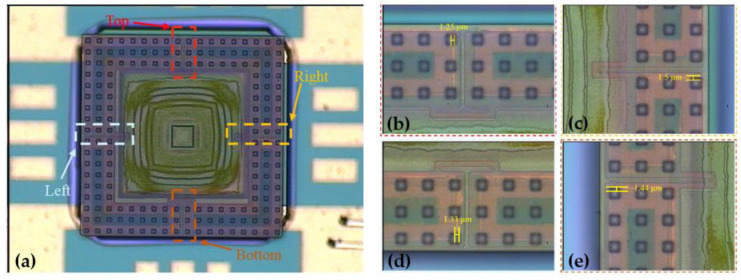
Microscopic view of transferred device and flip-chip bonding precision after the Au–Si eutectic flip-chip bonding process. (**a**) Microscopic view of transferred device; (**b**–**e**) Deviations of the top, right, bottom, and left metal electrode between the tactile sensor and the reroute substrate of approximately 1.25, 1.5, 1.33, and 1.44 μm, respectively.

The fabricated tactile sensor was first bonded to the reroute substrate by Fintech FinePlacer Lambda (Finetech GmbH & Co. KG, Berlin, Germany), and the temperature, force, and time of the flip-chip bonding process were 380 °C, 20 N, and 300 s, respectively. Then, the tactile sensor was transferred to the reroute substrate by applying a pulling force perpendicular to the bonded device, as shown in Figure 10. The tactile sensors with the designed stiction-contact structure areas from 2916 to 9000 ${\mu m}^{2}$ were transferred to the reroute substrate, and the optical microscopic view of the transferred devices with minimum and maximum stiction-contact structure areas are shown in Figure 10a,b, respectively. The metal bonding electrode deviations between the tactile sensor and the reroute substrate were measured to estimate the precision of the transferred device after the flip-chip bonding process, as shown in Figure 11. The metal electrode deviations of the top, right, bottom, and left are shown in Figure 11b–e, respectively. As shown in Figure 11b–e, the maximum measured deviation after the flip-chip bonding process is approximately 1.5 μm, which is sufficient for the proposed SETH integration process.

**Figure 12:**
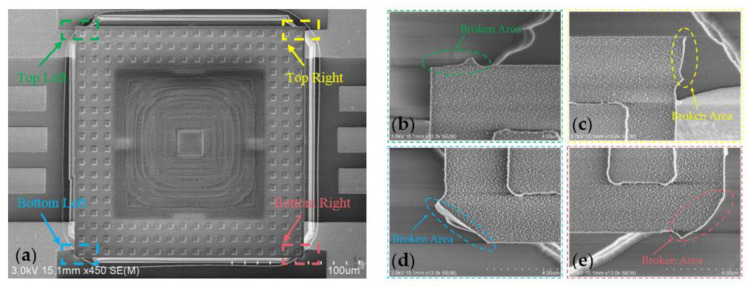
SEM view of the broken area of the temporary support structures of the tactile sensor. (**a**) SEM view of transferred device; (**b**–**e**) enlarged SEM views of the broken area shown in (**a**).

SEM was employed to analyze further the broken area of the temporary support beams of the tactile sensor after the transfer process. The SEM view of the broken area of the temporary support structures of the tactile sensor is shown in Figure 12a, and Figure 12b–d shows an enlarged SEM view of the broken area shown in Figure 12a. As Figure 12b–e show, the broken area was at the edge of the metal bonding electrode, and the silicon nitride diaphragm of the tactile sensor was not damaged after the transfer process.

**Figure 13:**
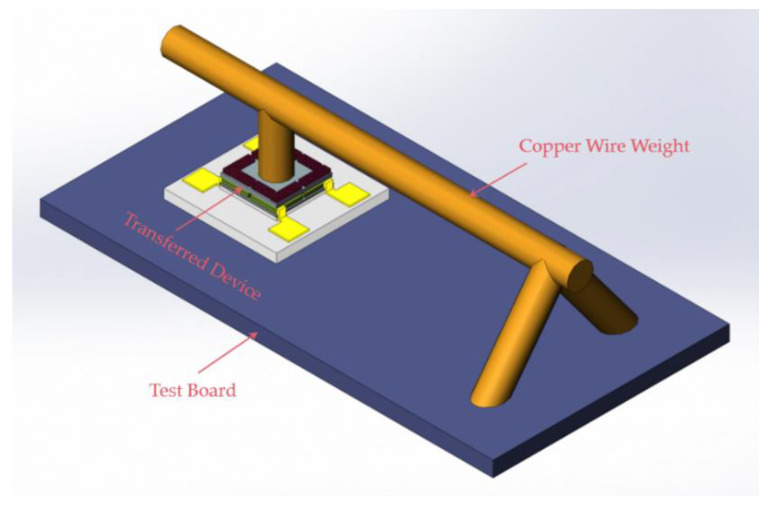
Schematic of the measurement principle of beam-shaped copper wire weights.

As Figure 11a and Figure 12a show, the size of the transferred tactile sensor is 180 μm × 180 μm × 1.2 μm, and the force area of the tactile sensor is only 120 μm × 120 μm × 1.2 μm. Moreover, the force application accuracy is extremely high because the designed full-scale force of the designed tactile sensor is only approximately 0.5 mN, and there is no suitable instrument to measure the sensitivity of the transferred tactile sensor directly. Therefore, different masses of the beam-shaped copper wire weights with a diameter of 85 μm are made to measure the sensitivity of the transferred tactile sensor, and the measurement principle of the beam-shaped copper wire weight is shown in Figure 13. As Figure 13 shows, one end of the beam-shaped copper wire weight is placed on the sensitive membrane of the transferred tactile sensor, and the other end is placed on the test stage. The force applied to the transferred tactile sensor is half of the mass of the beam-shaped copper wire weight.

**Figure 14:**
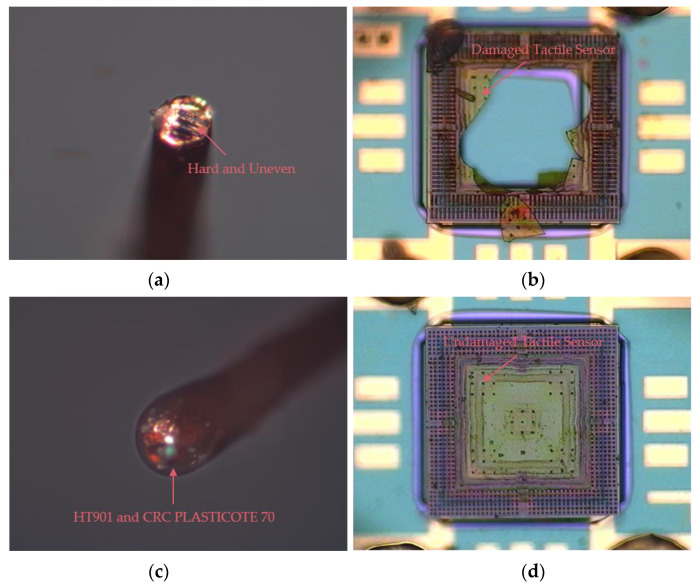
Tip of the copper wire weight. (**a**) The tip of the copper wire weight is hard and uneven; (**b**) The 1.2 μm-thick silicon nitride diaphragm is damaged during the test; (**c**) The layer of CRC PLASTICOTE 70 clear protective lacquer coated on the surface of the cured silicon adhesive sealant prevents the 1.2-μm-thick silicon nitride diaphragm from being damaged during the test; (**d**) The 1.2 μm-thick silicon nitride diaphragm is not damaged after the test.

**Figure 15:**
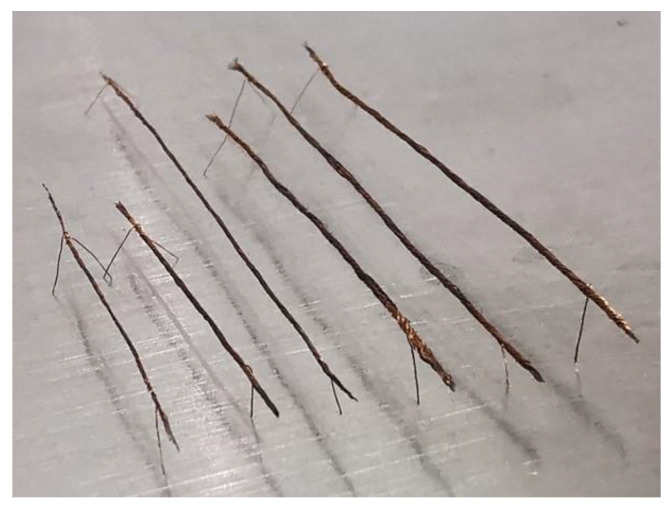
Different masses of manufactured beam-shaped copper wire weights.

As the tip of the copper wire weight is hard and uneven, which will damage the 1.2 μm-thick silicon nitride diaphragm of the tactile sensor when the copper wire weight is placed on the silicon nitride diaphragm, the surface of the copper wire weight is wrapped with a layer of HT901 silicon adhesive sealant and cured for 24 h at room temperature to make soften the tip of the copper wire weight. A layer of CRC PLASTICOTE 70 clear protective lacquer is then coated on the surface of the cured silicon adhesive sealant and cured for 12 h at room temperature to prevent the adhesive force of the silicon adhesive sealant from damaging the 1.2 μm-thick silicon nitride diaphragm, as shown in Figure 14. The masses of the different beam-shaped copper wire weights are measured using a Mettler Toledo AL104, with a readability of 0.1 mg, and the test results are shown in Table 1. The different masses of the manufactured beam-shaped copper wire weights are shown in Figure 15.

**Figure 16:**
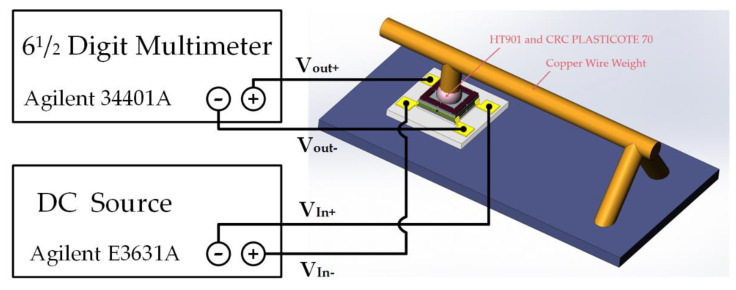
Schematic of the actual measurement setup.

**Figure 17:**
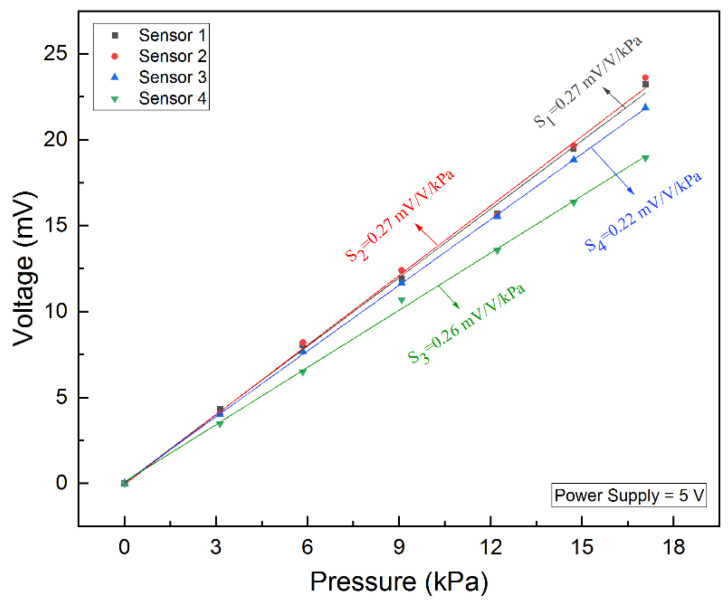
Sensitivity measurement result of the transferred tactile sensors.

The experimental setup used for measuring the sensitivity of the tactile sensor is shown in Figure 16. As shown in Figure 16, the input voltage of the Wheatstone bridge formed by the four piezoresistors of the transferred tactile sensor was first set to 5 V by Agilent E3631A (Agilent Tec., Santa Clara, CA, USA), and the corresponding output voltage value of the Wheatstone bridge was then recorded using Agilent 34401A when different masses of the beam-shaped copper wire weights were placed on the force area of the transferred tactile sensor under the microscope. The sensitivity measurement results of the tactile sensors are shown in Figure 17. As Figure 17 shows, the power supply of the transferred tactile sensor is 5 V, the output voltage of the Wheatstone bridge changes from 0 to 23.22 mV when the pressure applied to the transferred tactile sensors changes from 0 to 17.1 kPa, and the sensitivity of the four transferred tactile sensors are 0.22 mV/V/kPa, 0.26 mV/V/kPa, 0.27 mV/V/kPa and 0.27 mV/V/kPa, separately.

**Figure 18:**
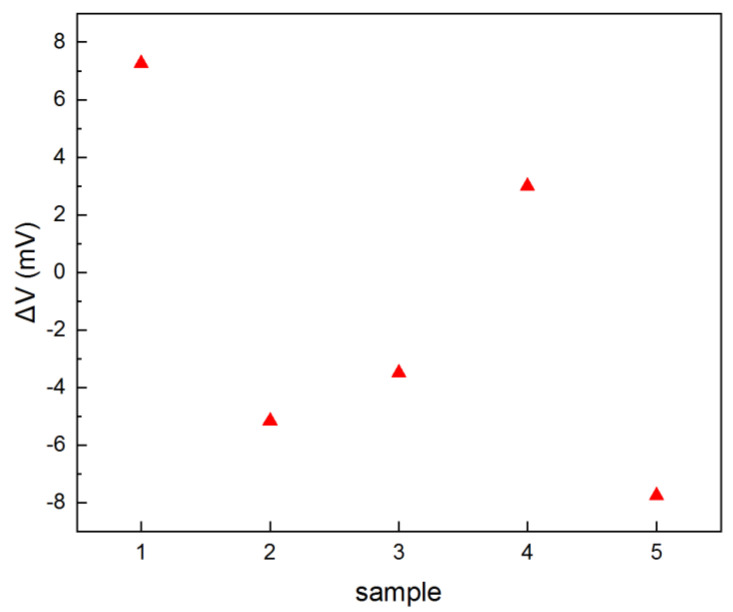
Output voltage difference of the Wheatstone bridge before and after the flip-chip bonding process.

Because the stress caused by the Au–Si eutectic flip-chip bonding process will affect the resistance of the four piezoresistors slightly, the output voltages of the Wheatstone bridge of the transferred tactile sensor were measured by an MPI TS2000-SE four-point probe before and after the flip-chip bonding process to estimate the stress caused by the Au–Si eutectic flip-chip bonding process, and the test result is shown in Figure 18. As Figure 18 shows, the output voltage difference of the Wheatstone bridge before and after the flip-chip bonding process was obtained from −7.76 to +7.25 mV, and the corresponding stress can be calculated from −5.83 kPa to +5.54 kPa, which indicated that the stress caused by the Au–Si eutectic flip-chip bonding process can be acceptable [32].

**Figure 19:**
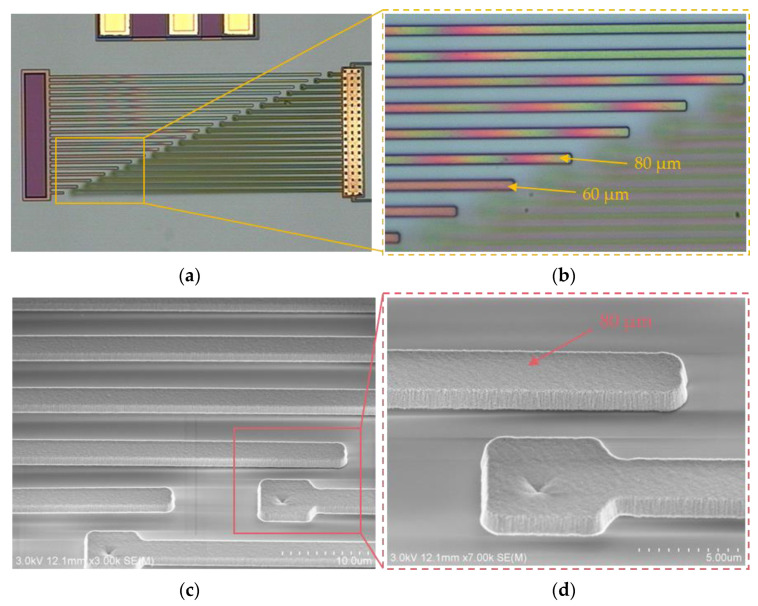
Optical microscopic and SEM views of the cantilever beams after drying at room temperature for 12 h. (**a**) Optical microscopic view of the silicon nitride cantilever beams; (**b**) enlarged optical microscope view of the silicon nitride cantilever beams shows that the tips of silicon nitride cantilever beams longer than 80 μm are bonded to the substrate; (**c**) SEM views of the silicon nitride cantilever beams; (**d**) enlarged SEM view of the silicon nitride cantilever beams shows that the tips of silicon nitride cantilever beams longer than 80 μm are bonded to the substrate.

The shear strength of the Au–Si eutectic bonding was tested using a Dage Series 4000 Bondtester (Nordson TEST & INSPECTION, Aylesbury, Buckinghamshire, UK), and the test structure is shown in Figure 4a. The area of the metal electrodes used for the Au–Si eutectic bond was approximately 0.34 ${\mathrm{mm}}^{2}$. The shear strength of the bonded test structure was approximately 30.74 MPa. To estimate the temporary bonding strength caused by stiction, the tips of silicon nitride cantilever beams with a length larger than 80 μm were then bonded to the substrate after the designed test structures shown in Figure 4b were placed in deionized (DI) water for 24 h and dried at room temperature for 24 h. The optical microscopic and SEM views of the cantilever beams are shown in Figure 19a–d, respectively. As the designed distance between the silicon nitride cantilever beams and substrate was 1 μm, using Equation (6), the temporary bonding strength can be calculated to be larger than 7.06 kPa and less than 22.31 kPa. Due to the bonding strength of the transferred tactile sensors are much larger than the temporary bonding strength caused by stiction, the tactile sensors can be easily transferred to the reroute substrate, and the yield of the transferred tactile sensors is as high as 90%.

## 5. Conclusions

This paper presented a novel method for CMOS-compatible batch transfer of tactile sensors using SETH process and Au–Si eutectic flip-chip bonding process, which allowed the tactile sensor and the CMOS devices to be manufactured separately to simplify the MEMS/CMOS integration process, improve the process reliability, and electrical performance, and reduce material constriction. The tactile sensor with a low-stress silicon nitride diaphragm was transferred to the reroute substrate successfully. The size of the transferred tactile sensor was as small as 180 μm × 180 μm × 1.2 μm, and the force area of the tactile sensor was only 120 μm × 120 μm × 1.2 μm. The tactile sensor was released and placed into deionized (DI) water for 24 h to bond the stiction-contact structures temporarily to the substrate through the stiction effect, thereby avoiding the damage and movement of the diaphragm of the tactile sensor during subsequent flip-chip bonding. The temporary bonding strength was calculated to be larger than 7.06 kPa and less than 22.31 kPa. The maximum misalignment of the flip-chip bonding process was approximately 1.5 μm. The stress caused by the Au–Si eutectic flip-chip bonding was from −5.83 to + 5.54 kPa. The tactile sensors were tested from 0 to 17.1 kPa, resulting in a sensitivity of 0.22 mV/V/kPa, 0.26 mV/V/kPa, 0.27 mV/V/kPa and 0.27 mV/V/kPa, separately. The shear strength of the bonded test structure was approximately 30.74 MPa and the yield of the transferred tactile sensors is as high as 90%.

## Figures and Tables

**Table 1:** Different masses of the beam-shaped copper wire weights measured by Mettler Toledo AL104.

Number	Measured Mass (mg)	Pressure Applied on Transferred Device (kPa)
1	9.2	3.1
2	17.2	5.9
3	26.7	9.1
4	35.9	12.2
5	43.3	14.7
6	50.2	17.1
